# Quantum stochastic transport along chains

**DOI:** 10.1038/s41598-020-66143-1

**Published:** 2020-06-25

**Authors:** Dekel Shapira, Doron Cohen

**Affiliations:** Department of Physics, Ben-Gurion University of the Negev, Beer-Sheva, 84105 Israel

**Keywords:** Quantum physics, Statistical physics, thermodynamics and nonlinear dynamics, Physics

## Abstract

The *spreading* of a particle along a chain, and its *relaxation*, are central themes in statistical and quantum mechanics. One wonders what are the consequences of the interplay between *coherent* and *stochastic* transitions. This fundamental puzzle has not been addressed in the literature, though closely related themes were in the focus of the Physics literature throughout the last century, highlighting quantum versions of Brownian motion. Most recently this question has surfaced again in the context of photo-synthesis. Here we consider both an infinite tight-binding chain and a finite ring within the framework of an Ohmic master equation. With added disorder it becomes the quantum version of the Sinai-Derrida-Hatano-Nelson model, which features sliding and delocalization transitions. We highlight non-monotonic dependence of the current on the bias, and a counter-intuitive enhancement of the effective disorder due to coherent hopping.

## Introduction

A prototype problem in Physics is the dynamics of a particle along chain that consists of sites. If the dynamics is coherent one expects to observe ballistic motion and Bloch oscillations^1^, while for stochastic dynamics one expects to see diffusion and drift. In the presence of disorder, additional fascinating effects emerge: an Anderson localization transition in the coherent problem, and a Sinai-Derrida sliding transition in the stochastic problem. In practical applications the particle can be an exiton^2–4^. Past literature regarding quantum spreading in chains^5–14^, including publications that address the photo-synthesis theme^15–24^, were focused mainly on the question how noise and dissipation affect coherent transport. In a sense, our interest is in the reversed question.

In the present work we assume *independent* mechanisms for stochastic asymmetric (dissipative) transitions, and for coherent hamiltonian (conservative) transitions. Such setup is not common: the standard models do not allow to tune on and off the two mechanisms independently. The question arises how the two mechanisms affect each other. Can we simply “sum up” known results for stochastic transport with known results for coherent motion in noisy environment? We shall see that the answer is not trivial. The main surprises come out once we take into account the presence of disorder (see below). An optional way to phrase the question: what is the quantum version of the prototype stochastic problem that is known in the literature as *random walk in random environment*. As we know from the above cited works, due to disorder, the stochastic dissipative dynamics is not merely a simple minded Brownian motion. We would like to know whether coherence has any implication on the predicted disorder-related crossovers.

**Figure 1:**
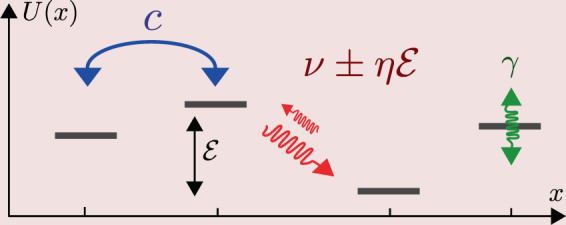
(1) Illustration of the model system. Each site of the chain is represented by a line segment positioned according to its $x$ coordinate and potential $U(x)$. Blue arrow labeled by $c$ represents the possibility for a coherent hopping between two sites. Red arrows represent bath induced stochastic transitions between two sites. The local bath that is responsible for the latter fluctuates with intensity $\nu$, and the induced transitions are asymmetric if $\eta =\nu /(2T)$ is non-zero (finite temperatures). Note that their ratio is exp$(\,-\,{\mathcal{E}}/T)$ in leading order. The green wiggle lines represent a local bath that induces fluctuations of intensity $\gamma$ of the on-site potential. Without the baths it is the Anderson model for coherent transport and localization in disordered chain. In the other extreme, if only the stochastic transitions are present, it is the Sinai-Derrida model for motion in random environment. The latter exhibits a sliding transition as the bias is increased, and an associated Hatano-Nelson delocalization transition once relaxation in a closed ring is considered.

We consider a chain whose sites are labeled by $x$. The particle, or the exiton, can move from site to site (near neighbor transitions only). The transitions are determined by two major parameters: the hopping frequency ($c$) that controls the coherent hopping; and the fluctuations intensity ($\nu$) that controls the environmentally-induced stochastic transitions. At finite temperature $T$ there is also a dissipation coefficient $\eta =\nu /(2T)$ that is responsible for the asymmetry of the stochastic transitions. On top we might have bias (${\mathcal{E}}$), on-site noisy fluctuations ($\gamma$), and different types of disorder. The model is illustrated in Fig. 1.

The dynamics is governed by a master equation for the probability matrix

1 $$\frac{d\rho }{dt}={\mathcal{L}}\rho =-\,i[{{\boldsymbol{H}}}^{(c)},\rho ]+({{\mathcal{L}}}^{({\rm{B}})}+{{\mathcal{L}}}^{({\rm{S}})})\rho $$

where the dissipators ${{\mathcal{L}}}^{({\rm{B}})}\propto \nu$ and ${{\mathcal{L}}}^{({\rm{S}})}\propto \gamma$ are due to the interaction with the environment. They are responsible for the stochastic aspect of the dynamics. The Hamiltonian ${{\boldsymbol{H}}}^{(c)}$ contains an on-site potential $U(x)$, and a sum over hopping terms $(c/2)|x\pm 1\rangle \langle x|$. Accordingly it takes the form

2 $${{\boldsymbol{H}}}^{(c)}\equiv U({\boldsymbol{x}})-c\,\cos ({\boldsymbol{p}})$$

where ***p*** is the momentum operator. The unit of length is the site spacing ($x$ is an integer), and the field is

3 $${{\mathcal{E}}}_{x}\equiv -\,(U(x+1)-U(x))$$

In the absence of stochastic terms, coherent transport in ordered chain leads to ballistic motion (without bias) and exhibits Bloch-oscillations (with bias). In disordered chain the spreading is suppressed due to Anderson-localization. The effect of noise and dissipation on coherent transport due to ${{\mathcal{L}}}^{({\rm{S}})}$ has been extensively studied. In the Caldeira-Leggett model^25,26^ the interaction is with homogeneous fluctuating environment, leading to Brownian motion with Gaussian spreading. If the interaction is with non-homogeneous fluctuating environment (short spatial correlation scale) the spreading is the sum of a decaying *coherent Gaussian* and a scattered *Stochastic Gaussian*^27^. The tight binding version of this model has been studied in^28^. It has been found that the decoherence and the stochastic-like evolution are dictated by different bands of the Lindblad ${\mathcal{L}}$-spectrum that correspond, respectively, to the dephasing and to the relaxation rates in NMR studies of two-level dynamics.

In the other extreme of purely stochastic dynamics, ignoring quantum effects, the disordered model, aka *random walk in random environment*, has been extensively studied by Sinai, Derrida, and followers^29–35^. Without bias the spreading becomes sub-diffusive, while above some critical bias the drift-velocity becomes finite, aka *sliding transition*. Strongly related is the transition from over-damped to under-damped relaxation that has been studied for a finite-size ring geometry^36,37^. The latter involves *delocalization transition* that has been highlighted for non-hermitian Hamiltonians in the works of Hatano, Nelson and followers^38–45^.

One should realize that the two extreme limits of coherent and stochastic spreading have to be bridged within the framework of a model that includes an ${{\mathcal{L}}}^{({\rm{B}})}$ term, not just an ${{\mathcal{L}}}^{({\rm{S}})}$ term. Furthermore, a proper modeling requires the distinction between two types of Master equations. In one extreme we have the *Pauli version*. Traditionally this version is justified by the secular approximation that assumes weak system-bath interaction. In the other extreme we have the *Ohmic version* that assumes short correlation time. The so called “singular coupling limit” can be regarded as an optional way to formalize the short correlation time assumption^46^. Clearly in the mesoscopic context it is more appropriate to adopt the Ohmic version, and regard the Pauli version of the dissipator as a formal approximation.

### Outline

**Figure 2:**
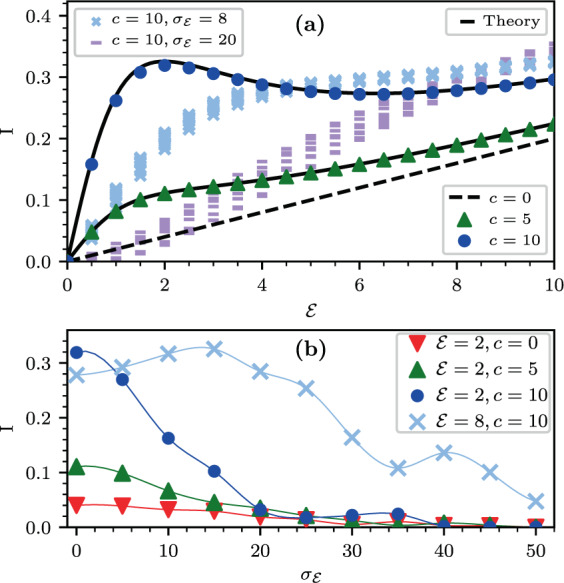
The NESS current for a biased chain, with and without disorder. (**a**) The NESS current as a function of ${\mathcal{E}}$. Black lines are based on Eq. (17) for clean system with $c=0,5,10$ and $\nu =1$ and $\eta =0.01$. Symbols are based on numerical determination of the NESS for a ring of $L=500$ sites. We display 10 independent realizations of the disorder for each value of disorder strength ${\sigma }_{{\mathcal{E}}}$, while $c=10$ is kept the same. (**b**) The average NESS current as a function of ${\sigma }_{{\mathcal{E}}}$ for ${\mathcal{E}}=2$. In the $c=10$ case also for ${\mathcal{E}}=8$. Thin-lines are a guide to the eye.

**Figure 3:**
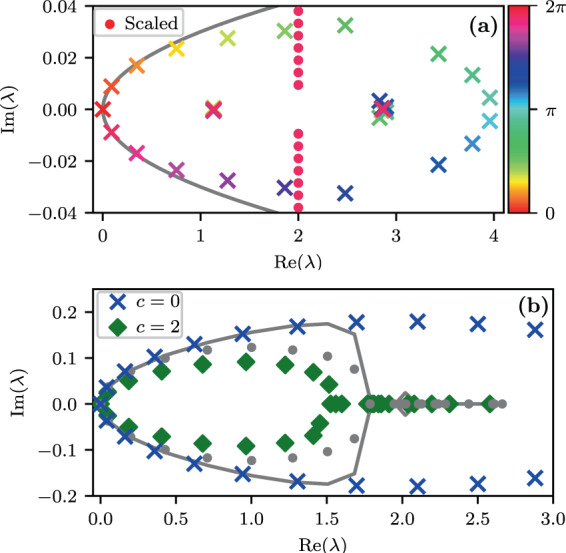
The Lindblad ${\mathcal{L}}$-spectrum for both non-disordered and disordered rings. (**a**) The spectrum for a non-disordered ring of $L=21$ sites. The eigenvalues that form an ellipse correspond to the stochastic-like relaxation modes. The eigenvalues that bunch together at $\lambda \sim 1,3$ are the $\mp$ over-damped decoherence modes. The other eigenvalues along ${\rm{Re}}(\lambda )=2$ correspond to under-damped decoherence modes (each point is in a fact a band of $L$ overlapping eigenvalues). The dashed gray-line is based on Eq. (18). For presentation purpose the eigenvalues marked with dot are scaled by a factor of 0.001 along the vertical axis. The colors indicate the $q$ of each eigenvalue. The parameters are $\nu =1,\eta =0.01,c=0.1,{\mathcal{E}}=0.5$. (**b**) The relaxation spectrum with disorder (decoherence modes are excluded). The spectrum for a chain with a given disorder is displayed, once with $c=0$ and once with $c=2$. The gray-circles and the gray-line are the three-band and one-band approximations for $c=2$. The other parameters are $L=31,{\mathcal{E}}=3,{\sigma }_{{\mathcal{E}}}=1.5,\nu =1,\eta =0.03$.

The model is presented in terms of an Ohmic master equation. The units of time are chosen such that the basic model parameters are $(c,{\mathcal{E}},\nu \equiv 1,\eta )$ and the strength of the disorder ${\sigma }_{{\mathcal{E}}}$. The interest is in the diffusion coefficient $D$, the ${\mathcal{E}}$-induced drift velocity $v$, the implied non equilibrium steady state (NESS) current $I\equiv (1/L)v$ for a ring of length $L$, and the associated Lindblad ${\mathcal{L}}$-spectrum. The latter is determined via ${\mathcal{L}}\rho =-\,\lambda \rho$, which provides both the relaxation-modes and the decoherence-modes. In particular we observe that the NESS current depends non-monotonically on the bias (Fig. 2a), and that surprisingly it can be enhanced by disorder (Fig. 2b). In a disordered ring, counter-intuitively, relaxation modes become over-damped if coherent transitions are switched on (Fig. 3).

## Results

### The model

The isolated chain is defined by the ***H***^(*c*)^ Hamiltonian Eq. (2), that describes a particle or an exiton that can hop along a one-dimensional chain whose sites are labeled by $x$. The field ${{\mathcal{E}}}_{x}$ might be non-uniform. For the average value of the field we maintain the notation ${\mathcal{E}}$, while the random component is distributed uniformly (box distribution) within $[\,-\,{\sigma }_{{\mathcal{E}}},{\sigma }_{{\mathcal{E}}}]$. We regard each pair of neighboring sites as a two-level system (Supplementary S1). Accordingly we distinguish between two types of terms in the master equation: those that originate from temporal fluctuations of the potential (dephasing due to noisy detuning), and those that are responsible to stochastic transition between the sites (incoherent hopping). The latter are implied by the replacement $(c/2)\mapsto (c/2)+f(t)$ at the pertinent bonds, where $f(t)$ is a bath operator that is characterized by fluctuation intensity $\nu$, and temperature $T$. Hence the system bath coupling term is $-\,{{\boldsymbol{W}}}_{x}\,f(t)$, where ${{\boldsymbol{W}}}_{x}=({{\boldsymbol{D}}}_{x}+{{\boldsymbol{D}}}_{x}^{\dagger })$ and ${{\boldsymbol{D}}}_{x}=|x+1\rangle \langle x|$. The baths of different bonds are uncorrelated, accordingly the bond-related dissipator takes the form

4 $${{\mathcal{L}}}^{({\rm{B}})}\rho =-\,\sum _{x}\left(\frac{\nu }{2}[{{\boldsymbol{W}}}_{x},[{{\boldsymbol{W}}}_{x},\rho ]]+\frac{\eta }{2}i[{{\boldsymbol{W}}}_{x},\{{{\boldsymbol{V}}}_{x},\rho \}]\right)$$

where $\eta =\nu /(2T)$ is the friction coefficient, and

5 $${{\boldsymbol{V}}}_{x}\equiv i[{{\boldsymbol{H}}}^{(c)},{{\boldsymbol{W}}}_{x}]$$

The friction terms represent the response of the bath to the rate of change of the ***W***_*x*_. Note that for getting the conventional Fokker-Planck equation the system-bath coupling term would be $-{\boldsymbol{x}}\,f(t)$, and ***V*** would become the velocity operator. Here we assume interaction with local baths that in general might have different temperatures. See Methods for some extra technical details regarding the master equation, the nature of the disorder, and the handling of the periodic boundary conditions for the ring configuration.

### Pauli-type dynamics

For pedagogical purpose let us consider first a uniform non-disordered ring without coherent hopping. Furthermore, let us adopt the simplified Pauli-like version of the dissipator (see Methods). Consequently the dynamics of the on-site probabilities ${p}_{x}\equiv {\rho }_{x,x}$ decouples from that of the off-diagonal terms. Namely, one obtains for the probabilities a simple rate equation, where the transition rates between sites are

6 $${w}^{\pm }=\nu \pm \eta {\mathcal{E}}$$

in agreement with Fermi-golden-rule (FGR). Note that in leading order $[{w}^{-}/{w}^{+}]\approx \exp (\,-\,{\mathcal{E}}/T)$ as expected from detailed balance considerations. It follows that the drift velocity and the diffusion coefficient are:

7 $$v=({w}^{+}-{w}^{-})=2\eta {\mathcal{E}}$$

8 $$D=\frac{1}{2}({w}^{+}+{w}^{-})=\nu $$

Consequently one finds two distinct sets of modes: the stochastic-like relaxation modes that are implied by the rate equation for the probabilities, and off-diagonal decoherence modes. The latter share the *same* decay rate ${\gamma }_{0}={w}^{+}+{w}^{-}+\gamma$, where $\gamma$ stands for optional extra off-diagonal decoherence due to on-site fluctuations. An evolving wavepacket (Supplementary S3) will decompose into coherent decaying component that is suppressed by factor ${e}^{-{\gamma }_{0}t}$, and an emerging stochastic component that drifts with velocity $v$ and diffuse with coefficient $D$.

### Full Ohmic treatment

The state of the particle in the standard representation is given by ${\rho }_{x}(r)\equiv \langle x|\rho |x+r\rangle$. The master equation, Eq. (1) with Eq. (4), couples the dynamics of the on-site probabilities ${p}_{x}\equiv {\rho }_{x}(0)$ to that of the off-diagonal elements ${\rho }_{x}(r\ne 0)$. The generator ${\mathcal{L}}$ can be written as a sum of several terms (Supplementary S5):

9 $${\mathcal{L}}={\mathcal{E}}{{\mathcal{L}}}^{({\mathcal{E}})}+c{{\mathcal{L}}}^{(c)}+\nu {{\mathcal{L}}}^{(\nu )}+\eta {\mathcal{E}}{{\mathcal{L}}}^{(\tilde{{\mathcal{E}}})}+\eta c{{\mathcal{L}}}^{(\tilde{c})}$$

Each term is a super-matrix that operates on the super vector ${\rho }_{x}(r)$. The first two terms ${{\mathcal{L}}}^{(c)}$ and ${{\mathcal{L}}}^{({\mathcal{E}})}$ arise from the Hamiltonian Eq. (2). The ${{\mathcal{L}}}^{(\nu )}$ term arise from the first term of Eq. (4), which represent noise-induced transitions. The remaining two friction-terms (proportional to $\eta$) arise from Eq. (5), and correspond to the two terms in the Hamiltonian.

**Figure 4:**
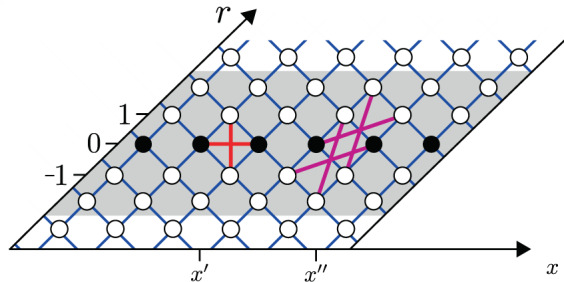
Diagrammatic representation of the couplings in the master equation. A diagonal strip of the probability matrix ${\rho }_{x}(r)$ is illustrated. The diagonal elements ${p}_{x}={\rho }_{x}(0)$ are represented by filled circles, and the off-diagonal terms by empty circles. The Lindblad generator ${\mathcal{L}}$ induces “transitions” between the elements. The blue grid lines indicate $c$-induced couplings. The other couplings within $|r|\le 2$, indicated by red and purple, are due to a local bath. For presentation purpose (to avoid a crowded set of lines) the red couplings that originate from $\nu {{\mathcal{L}}}^{(\nu )}$ and $\eta {\mathcal{E}}{{\mathcal{L}}}^{(\tilde{{\mathcal{E}}})}$ assume that the local bath is positioned at bond *x*′, while purple couplings that originate from $\eta c{{\mathcal{L}}}^{(\tilde{c})}$ assume that the bath is positioned at bond *x*″.

A schematic representation of ${\rho }_{x}(r)$ and the couplings is given in Fig. 4. The coherent hopping that is generated by ${{\mathcal{L}}}^{(c)}$ couples ${\rho }_{x}(r)$ to ${\rho }_{x}(r\pm 1)$ and to ${\rho }_{x+1}(r\pm 1)$, while ${{\mathcal{L}}}^{({\mathcal{E}})}$ contribute “on-site” potential. The noise operator $\nu {{\mathcal{L}}}^{(\nu )}$ include the Pauli-terms that were discussed previously, and an additional term that couples the $r=\pm \,1$ elements. Together with the friction operator $\eta {\mathcal{E}}{{\mathcal{L}}}^{(\tilde{{\mathcal{E}}})}$, the Pauli terms induce the asymmetric $x\pm 1$ stochastic transitions of Eq. (6) along $r=0$. The second friction term ${{\mathcal{L}}}^{(\bar{c})}$ consists of non-Pauli terms that allow extra ${{\mathcal{L}}}^{(c)}$-type couplings, and in particular extra $x\pm 2$ transitions within the strip $|r|=0,1,2$.

### The spectrum for a non-disordered ring

For a non-disordered ring the super-matrix ${\mathcal{L}}$ is invariant under $x$-translations, and therefore we can switch to a Fourier basis where the representation is $\rho (r;q)$. Due to Bloch theorem, the matrix decompose into $q$-blocks in this basis. Thus in order to find the eigenvalues ${\lambda }_{q,s}$ and the corresponding eigenmodes we merely have to handle a one dimensional tight binding $|r\rangle$ lattice. See Methods. A representative spectrum is provided in Fig. 3a. Consider first the $q=0$ eigenstates. For $q=0$ the $c$-dependent couplings are zero. For infinite temperature ($\eta =0$) the only non-zero coupling is between $|r=\pm \,1\rangle$ due to a non-Pauli term in Eq. (32). Consequently the $q=0$ block contains the NESS $|r=0\rangle$ (which is merely the identity matrix in the standard basis), along with a pair of non-trivial decoherence modes $|\pm \rangle$, and a set of uncoupled decoherence modes $|r=\pm \,2,\pm \,3,\ldots \rangle$. The corresponding ${\lambda }_{q,s}$ eigenvalues (for $\eta =0$) are:

10 $${\lambda }_{0,0}=0\,({\rm{NESS}})$$

11 $${\lambda }_{0,\pm }=2\nu \pm \sqrt{{\nu }^{2}-{{\mathcal{E}}}^{2}}$$

12 $${\lambda }_{0,s}=2\nu +i{\mathcal{E}}s,\,(s=\pm \,2,\pm \,3,\ldots )$$

The $|\pm \rangle$ modes become over-damped for small bias, while the $|s| > 1$ decoherence modes are always under-damped. Considering the $q$ dependence of the eigenvalues ${\lambda }_{q,s}$ we get several bands, as illustrated in Fig. 3a. Our interest below is in the relaxation modes that are associated with ${\lambda }_{q\mathrm{,0}}$, and determine the long time spreading.

### The NESS

At finite temperature ($\eta  > 0$) there are extra couplings that lead to a modified NESS. In leading order the NESS eigenstate is $|0\rangle +{\alpha }_{0}|1\rangle +{\alpha }_{0}^{\ast }|-1\rangle$ with

13 $${\alpha }_{0}=\frac{3\nu -i{\mathcal{E}}}{3{\nu }^{2}+{{\mathcal{E}}}^{2}}\eta c$$

Reverting back to the standard representation we get

14 $${\rho }^{({\rm{NESS}})}=\frac{1}{L}(1+{\alpha }_{0}{e}^{+ip}+{\alpha }_{0}^{\ast }{e}^{-ip})$$

From this we can deduce the steady state momentum distribution [S5], namely, $p(k)\equiv \langle k|{\rho }^{({\rm{NESS}})}|k\rangle$. The result in leading order is

15 $$p(k)\propto \exp \left[\frac{2\eta c}{3{\nu }^{2}+{{\mathcal{E}}}^{2}}(3\nu \,\cos (k)+{\mathcal{E}}\,\sin (k))\right]$$

For ${\mathcal{E}}=0$ this expression is consistent with the canonical expectation exp(−*β****H***^(*c*)^).

### The current

For non-zero field (${\mathcal{E}}\ne 0$) the NESS momentum distribution is shifted. The expression for the current operator is complicated (Supplementary S2), but the net NESS current comes out a simple sum of stochastic and coherent terms:

16 $${I}_{x}=\frac{1}{L}(({w}_{x}^{+}-{w}_{x}^{-})-c\,{\rm{Im}}({\alpha }_{0}))$$

17 $$\,=\frac{1}{L}\left[1+\frac{{c}^{2}}{6{\nu }^{2}+2{{\mathcal{E}}}^{2}}\right]\,2\eta {\mathcal{E}}\equiv \frac{1}{L}v$$

We shall further illuminate the physical significance of the second term below. In contrast with the stochastic case, the drift current might be non-monotonic in ${\mathcal{E}}$, see Fig. 2a. Furthermore, there is a convex range where the second derivative of $I({\mathcal{E}})$ is positive.

The *convexity* of the current in some ${\mathcal{E}}$ range, implies a counter intuitive effect: current may become larger due to disorder. The argument goes as follows: Assume that the sample is divided into two regions, such that ${{\mathcal{E}}}_{x}$ is constant in each region, but slightly smaller (larger) than ${\mathcal{E}}$ in the first (second) region. Due to the convex property it is implied that the current will be larger. Extending this argument for a general non-homogeneous (i.e. disordered) field, with the same average bias ${\mathcal{E}}$, we expect to observe a *larger* NESS current. This is indeed confirmed in Fig. 2a, while additional examples are given and discussed quantitatively in Supplementary S6.

### The Diffusion

An optional way to derive Eq. (17) is to expand ${\lambda }_{q\mathrm{,0}}$ in $q$, to obtain $v$. The second order term gives the diffusion coefficient. Namely,

18 $${\lambda }_{q,0}=ivq+D{q}^{2}+O({q}^{3})$$

It is therefore enough to determine ${\lambda }_{q\mathrm{,0}}$ via second order perturbation theory with respect to the $q=0$ eigenstates. To leading order in $\eta$, a lengthy calculation leads to a result that is consistent with the Einstein relation, namely

19 $$\frac{v}{D}=\frac{{\mathcal{E}}}{T},\,[{\rm{valid}}\,{\rm{in}}\,{\rm{leading}}\,{\rm{order}}]\,$$

Thus, to leading order, $D$ is given by Eq. (8), multiplied by the expression in the square brackets in Eq. (17). We see that with coherent transitions, for zero bias, this expression takes the form $D=\nu +{D}_{\ell }$, where ${D}_{\ell }={c}^{2}/(6\nu )$. The latter can be interpreted as a Drude-type result ${D}_{\ell }={\ell }^{2}/\tau$, with relaxation time $\tau \sim \mathrm{1/}\nu$ and mean free path $\ell \sim c\tau$. A similar expression has been obtained in^5,28^ for a chain with noisy sites. In the other extreme of large bias Eq. (8) implies that ${D}_{\ell }=(1/2)|c/{\mathcal{E}}{|}^{2}\nu$. This result, like the Drude result, can be regarded as coming from FGR transitions. But now the transitions are between Bloch site-localized states. Namely, we have hopping between neighboring sites ($\ell \sim 1$), with rate of the transitions ($\mathrm{1/}\tau$) that is suppressed by a factor $|c/{\mathcal{E}}{|}^{2}$. The suppression factor reflects the first-order-perturbation-theory overlap of Bloch-localized wavefunctions. To summarize: we can say that ${D}_{\ell }$ exhibits a crossover from Drude-type transport to hopping-type transport as the field ${\mathcal{E}}$ is increased.

In fact we can proceed beyond leading order, and calculate $D$ up to second order in $\eta$, see (Supplementary S5). Here we cite only the zero bias result:

20 $$D=\left[1+\frac{{c}^{2}}{6{\nu }^{2}}-\frac{{c}^{2}}{4{T}^{2}}\right]\,\nu $$

In view of the Drude picture this result is surprising. Namely, one would expect ${D}_{\ell }\sim {c}^{2}\tau$ to be replaced by ${D}_{\ell }\sim \langle {v}^{2}\rangle \tau$, and hence one would expect the replacement ${c}^{2}\mapsto (1-[1/8]{(c/T)}^{2}){c}^{2}$ due to the narrowing of the momentum distribution, see Methods. However the current result indicates that the leading correction is related to a different mechanism. Indeed, using a semiclassical perspective, the coupling to the bath involves a $\cos (p)$ factor, see Methods. The zero order diffusion with rate $\nu$ arises due to stochastic term in the equation of motion for $\dot{x}$ that involves a $\sin (p)$ factor, Consequently, due to thermal averaging, $\nu \mapsto (1-[1/8]{(c/T)}^{2})\nu$, which explains, up to a factor of 2, the third term in Eq. (20). We have repeated this calculation also for a Caldeira-Leggett dissipator, and also for an ${{\mathcal{L}}}^{({\rm{S}})}$ dissipator. For the former the expected $(c/T)$ correction to ${D}_{\ell }$ appears, but has a different numerical factor, while for the latter the correction comes out with an opposite sign. We can show analytically that the discrepancies are due to the modification of the correlation time^47^.

### Disordered ring

The so called stochastic field ${{\mathcal{E}}}_{x}/T$ is responsible for the asymmetry of the incoherent transitions. Following Sinai we assume that it has a random component that is (say) box-distributed. From the works of Sinai and Derrida^30–32^ we expect a sliding transition as ${\mathcal{E}}/T$ exceeds a critical value of order ${({\sigma }_{{\mathcal{E}}}/T)}^{2}$. Strongly related is the delocalization transition^36,38–40,43,44^ for which the critical value is smaller by a numerical factor. Disregarding this factor we expect

21 $${{\mathcal{E}}}_{c}\approx \frac{1}{T}{\sigma }_{{\mathcal{E}}}^{2}$$

In the purely stochastic model, for ${\mathcal{E}} > {{\mathcal{E}}}_{c}$ the relaxation is expected to be under-damped due to a delocalization transition that leads to the appearance of complex eigenvalues at the vicinity of $\lambda =0$.

The question arises how this transition is affected by quantum coherent hopping. The naive expectation would be to witness a smaller tendency for localization in the relaxation-spectrum because we add coherent bypass that enhances the transport. But surprisingly the numerical results of Fig. 3b show that the effect goes in the opposite direction: for non-zero $c$, some eigenvalues become real, indicating stronger effective disorder.

### Enhanced effective disorder

We turn to provide an explanation for observing enhanced effective disorder due to coherent hopping. On the basis of the non-disordered ring analysis, the relaxation modes occupy mostly the $|r|=0,1$ diagonals of $\rho$, and therefore it makes sense to exclude couplings to the higher diagonals. We verify that this does not change the qualitative picture in Fig. 3b (gray vs green symbols). The effect of the $|r|=1$ band is to introduce virtual coherent transitions between diagonal elements (Supplementary S7). Hence we end up with an effective single-band stochastic equation with transition rates

22 $${w}_{x}^{\pm }=\nu +{\nu }_{x}\pm \eta {{\mathcal{E}}}_{x}\equiv {w}_{x}\,\exp (\,\pm \,{\tilde{{\mathcal{E}}}}_{x})$$

23 $${\nu }_{x}=\frac{{c}^{2}}{2}\frac{\nu -\lambda }{{(2\nu -\lambda )}^{2}+{{\mathcal{E}}}_{x}^{2}-{\nu }^{2}}$$

The disorder that is associated with ${\nu }_{x}$ is hermitian, namely, it does not spoil the symmetry of the transitions, it merely implies that the we have a tight binding model with random couplings that have some dispersion ${\sigma }_{\perp }^{2}\equiv {\rm{Var}}({w}_{x})\propto {c}^{4}$. This is known as *resistor network* (RN) disorder. In contrast, the $\pm \eta {{\mathcal{E}}}_{x}$ term induces *asymmetric* transitions. This type of non-hermitian Sinai-type disorder is characterized by the dispersion ${\sigma }_{\parallel }^{2}\equiv {\rm{Var}}({\tilde{{\mathcal{E}}}}_{x})$. The latter translates after non-hermitian gauge transformation to (hermitian) diagonal disorder, with ill-defined boundary conditions. The procedure to handle both types of disorder has been discussed in^36^ following^39^. One defines an hermitian RN matrix by setting ${\tilde{{\mathcal{E}}}}_{x}=0$ in Eq. (22). The RN matrix has a real spectrum with eigenstates that are characterized by inverse localization length that is dominated by the RN-disorder, namely, $\kappa (\lambda )\propto {\sigma }_{\perp }^{2}\lambda$. Adding back the field ${{\mathcal{E}}}_{x}$, the eigenstates remain localized (with real eigenvalues) only in regions where localization is strong enough, that is, $\kappa (\lambda ) > {\mathcal{E}}/T$. Estimating ${\sigma }_{\perp }$, see Supplementary S7 for an explicit expression, we deduce that the additional RN disorder is responsible for the observed numerical result.

### The Wannier-Stark Ladder

**Figure 5:**
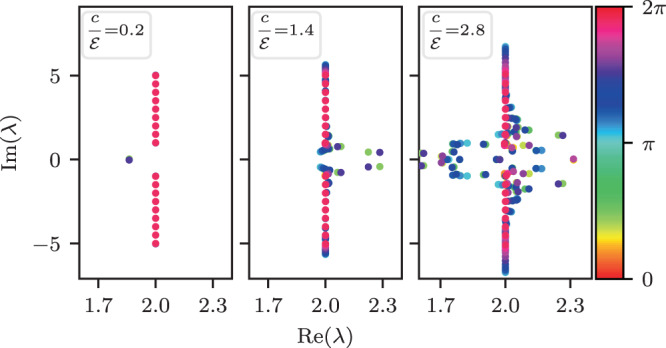
Blurring of the Wannier-Stark Ladder. Here the parameters are $\nu =1,\eta =0.03,{\mathcal{E}}=0.5$ and $L=21$. The parameter $c/{\mathcal{E}}$ determines the localization range of the Bloch eigenstates. The small $q$ modes are weakly coupled by $\nu$ and therefore maintain the ${\mathcal{E}}$ spacing as implied by Eq. (12).

We shift our attention to the full Lindblad spectrum. In the absence of coupling to the bath, the eigenstates of the Hamiltonian Eq. (2) are Bloch localized. Each eigenstate occupies a spatial region ~*c*/${\mathcal{E}}$, and the corresponding eigen-energies form a ladder with spacing ${\mathcal{E}}$, that reflects the frequency of the Bloch oscillations. Weak coupling to the bath leads to damping of the Bloch oscillations. This is reflected by the Lindblad spectrum. For the $q=0$ modes we have obtained Eq. (12), where we see that the eigenvalues acquire a real part, but maintain the ladder structure. But for non-zero $q$ the ${{\mathcal{L}}}^{(c)}$ term couples the modes to the perturbation that is created at the $|r| < 2$ region by ${{\mathcal{L}}}^{(\nu )}$, see Eqs. (31) and (32) of the Methods. This results in a deformation of the ladder. Namely, the ladder consists of bands, and the number of bands that are deformed equals the Bloch localization length. See Fig. 5.

### Regime diagram

We would like to place our results in the context of the vast quantum dissipation literature. The prototype model of Quantum Brownian Motion (QBM), aka the Caldeira-Leggett model, involves coupling to a single bath that exerts a fluctuating homogeneous field of force. In the classical framework it leads to the standard Langevin equation

24 $${\rm{m}}\ddot{x}={\mathcal{E}}-\eta \dot{x}+f(t)$$

and Eq. (1) becomes the standard Fokker Planck equation. In the tight-binding framework we have the identification ${\rm{m}}\,\mapsto \,\mathrm{1/(}c{a}^{2})$, where $a$ is the lattice constant. The standard QBM model features a single dimensionless parameter, the scaled inverse temperature $\beta$, which is the ratio between the thermal time $\mathrm{1/}T$ and damping time ${\rm{m}}/\eta$. In the lattice problem we can define two dimensionless parameters

25 $$\alpha =\frac{1}{2\pi }\eta {a}^{2}\,=\,{\rm{s}}{\rm{c}}{\rm{a}}{\rm{l}}{\rm{e}}{\rm{d}}\,{\rm{f}}{\rm{r}}{\rm{i}}{\rm{c}}{\rm{t}}{\rm{i}}{\rm{o}}{\rm{n}}\,$$

26 $$\theta =\frac{T}{c}=\frac{\nu }{2\eta c}\,=\,{\rm{s}}{\rm{c}}{\rm{a}}{\rm{l}}{\rm{e}}{\rm{d}}\,{\rm{t}}{\rm{e}}{\rm{m}}{\rm{p}}{\rm{e}}{\rm{r}}{\rm{a}}{\rm{t}}{\rm{u}}{\rm{r}}{\rm{e}}$$

Accordingly $\beta =\alpha /\theta$. Note that in our model we set the units such that $a=1$, hence, disregarding $2\pi$ factor, our scaled friction parameter $\eta$ is the same as $\alpha$.

The standard analysis of QBM^48^ reveals that quantum-implied memory effects are expressed in the regime $\beta \gg 1$, where a transient log(*t*) spreading is observed in the absence of bias, followed by diffusion. Most of the quantum dissipation literature, regarding the two-site spin-boson model^49^ and regarding multi-site chains^50,51^, is focused in this low temperature regime, where significant deviations from the classical predictions are observed for large $\alpha$ of order unity. In contrast, our interest is in the $\alpha ,\beta \ll 1$ regime.

**Figure 6:**
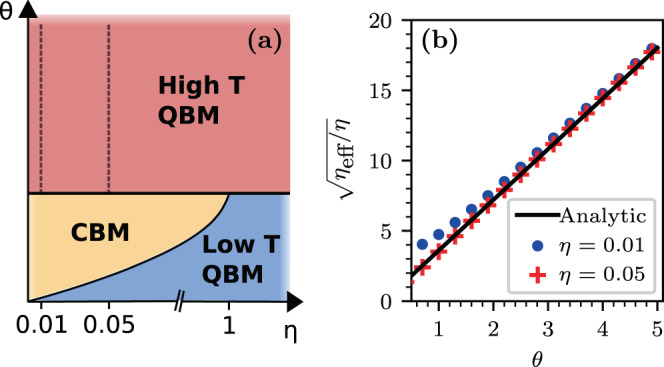
The Brownian Motion regime diagram. (**a**) The various regions in the $(\eta ,\theta )$ diagram are indicated. We distinguish between the Classical-like Brownian Motion (CBM) region; the low-temperature QBM region where memory effects dominates; and the high-temperature QBM region that has been discussed in this article. The Lindblad correction to the Ohmic master equation is negligible above the solid $\theta \sim 1$ line. (**b**) The effective friction coefficient ${\eta }_{{\rm{eff}}}$ of Eq. (27) is determined numerically along the two dashed lines of panel (a), and compared with the analytical prediction of Eq. (17). The parameters are $\nu =1,{\mathcal{E}}=0.5$ and $L=500$. For lower $\theta$, the Lindblad correction becomes important (not shown).

Our $(\eta ,\theta )$ regime diagram Fig. 6 is roughly divided into two regions by the line $\theta \sim 1$. Along this line the thermal de-Broglie wavelength of the particle is of order of the lattice constant, hence it bears formal analogy to the analysis of QBM in cosine potential^52^, where it marks the border to the regime where activation mechanism comes into action. In our tight binding model we have a single band, hence transport via thermal activation is not possible. Rather, in the $\theta  > 1$ regime, where $T\gg c$, the momentum distribution within the band is roughly flat, and the drift is dictated by Eq. (17), that is,

27 $$v=2\eta {\mathcal{E}}+\frac{1}{{\eta }_{{\rm{eff}}}}{\mathcal{E}}$$

where ${\eta }_{{\rm{eff}}}\approx 12{\theta }^{2}\eta$ for weak field. The low temperature regime $\theta \ll 1$ has not been addressed in this work, because the Ohmic master equation fails to reproduce canonical equilibration in this regime (see Methods). Still we would like to illuminate what we get (not presented) if this aspect is corrected. In the regime $\theta \ll 1$ the momentum distribution becomes much narrower (only low energy momenta are populated) and therefore ${\eta }_{{\rm{eff}}}\sim \eta$ as implied by Eq. (24). This we call Classical-like Brownian motion (CBM) regime. Once the coupling to the bath is not the simple $x$-coupling of the Caldeira-Leggett model, a numerical prefactor is expected (see Methods for detailed argument).

## Discussion

There is a rich literature regarding Quantum Brownian motion (see^48,53–55^ and references within). In the condensed matter literature it is common to refer to the Caldeira-Leggett model^25,26^, where the particle is linearly coupled to a bath of harmonic oscillators that mimic an Ohmic environment. Some works study the motion of a particle in a periodic potential, possibly with bias, aka washboard potential^51,52,56^, while other refer to tight binding models^6,50,57^ as in this work. The focus in those papers is mostly on non-Markovian effects: at low temperatures the fluctuations are not like “white noise”, and are dominated by a high frequency cutoff ${\omega }_{c}$. Consequently the handling of long-time correlations becomes tricky. In this context the low temperature dependence of the diffusion and the mobility is modified for $\alpha  > 1/2$ and $\alpha  > 1$.

The line of study in the above models has assumed that the fluctuations that are induced by the bath are uniform in space. In some other works the dynamics of a particle that interacts with local baths has been considered. In such models the fluctuations acquire finite correlation length in space^5,7–14,27,28^. The extreme case, as in this work, are tight binding models where the coupling is to uncorrelated baths that seat on different sites or bonds. Studies in this context assume bath that are connected at the end points^58^, or baths that act as noise source^5^. Reference ^28^ has analyzed the spectral properties for a chain with noisy sites, ref. ^9^ has considered colored noise sources strongly coupled to each site, ref. ^59^ has considered noisy transitions on top of coherent transitions, ref. ^60^ has considered transport in the presence of dephasing and disorder, ref. ^11^ has considered numerically transport properties of a noisy system with static disorder, and^61^ has addressed some bounds in the absence of disorder. The basic question of transport in a tight-binding models has resurfaced in the context of excitation transport in photosynthetic light-harvesting complexes^15–24^.

It is quite surprising that all of the above cited works have somehow avoided the confrontation with themes that are familiar from the study of stochastic motion in random environment. Specifically we refer here to the extensive work by Sinai, Derrida, and followers^29–35^, and the studies of stochastic relaxation^36,37^ which is related to the works of Hatano, Nelson and followers^36–45^. Clearly we have here a gap that should be bridged.

In this article we have studied transport properties along a chain taking into account several themes that have not been combined in past studies: **(a)** The baths on different bonds are not correlated in space; **(b)** The baths are not just noise - the temperature is high but finite; **(c)** Without coherent hopping it is the Sinai-Derrida-Hatano-Nelson model which exhibits sliding and delocalization transitions; **(d)** Without baths it is a disordered chain with Anderson localization; **(e)** The bias might be large such that Bloch dynamics is reflected.

The “small” parameter in our analysis is the inverse temperature. The following observations have been highlighted: **(1)** The NESS current is the sum of stochastic and quasi-coherent terms; **(2)** It displays non-monotonic dependence on the bias, as shown in Fig. 2, due to crossover from Drude-type to hopping-type transport; **(3)** Disorder may increase the current due to convex property; **(4)** The interplay of stochastic and coherent transition is reflected in the Lindblad spectrum; **(5)** In the presence of disorder the quasi-coherent transitions enhance the localization of the relaxation modes. Thus, with regard to the Sinai-Derrida sliding transition, and the strongly related Hatano-Nelson delocalization transition, we find that adding coherent transitions “in parallel” have in some sense opposite effects: on the one hand they add bypass for the current (point (1) above), but on the other hand they enhance the tendency towards localization (point (5) above). Some of our results might be relevant to studies of optimal transport efficiency and the quantum Goldilocks effect^9^.

## Methods

### Master equation for disordered chain

A pedagogical presentation of the procedure for the construction of an Ohmic master equation for a two site system, and then for a chain, is presented in Supplementary S1. Each bond has a different bath, and therefore can experience different temperature and friction. Accordingly we can have disorder that originates either from the Hamiltonian (say random ${{\mathcal{E}}}_{x}$ as assumed in the main text), or from having different baths (random ${\nu }_{x}$ or random ${\eta }_{x}$). This extra disorder can be incorporated in a straightforward manner, and does not affect the big picture.

### Thermalization

The Ohmic master equation for a Brownian particle, if the coupling to the bath were $-{\boldsymbol{x}}f(t)$, is the standard Fokker-Plank equation. It leads to canonical thermal state for any friction and for any temperature. This is not the case for a discrete two level system. The agreement with the canonical result is guaranteed only to first order in $\eta$. This is reflected in Eq. (6). The same applies for a chain. Note that in this sense the Ohmic approximation is very different from the secular or Pauli approximation^62^, or specially constructed Davies Liovillian^63^, that guarantee canonical thermalization.

It is important to identify the “small parameter” that controls the deviation from canonical thermalization. The standard coupling via ***x*** induce transitions between neighboring momenta, and therefore the small parameter is Δ/*T*, where the level spacing Δ goes to zero in the $L\to \infty$ limit. But for local baths, the coupling is to $\delta ({\boldsymbol{x}}-{x}_{\alpha })$ scatterers, that create transitions to all the levels within the band. Therefore the small parameter in the absence of bias is $c/T$. This assertion is confirmed numerically by inspection of the equilibrium momentum distribution $p(k)$, see Supplementary S5. We conclude that in the regime of interest ($\theta  > 1$) the Ohmic master equation can be trusted, while for lower temperatures we have to “correct” it. For the two site system the “corrected” equation is the Bloch equation, where the ratio of the rates is in agreement with detailed balance (not just in leading order in $\eta$). For a chain, we cannot justify the secular approximation, and therefore the correction procedure is ill defined.

### The friction coefficient

In the Caldeira-Leggett model for Brownian particle, with interaction term $-{\boldsymbol{x}}f(t)$, the bath induced fluctuations $f(t)$ are determined by a coupling constant $\eta$, and by the bath temperature $T$, such that at high temperatures the intensity of the fluctuations is $\nu =2\eta T$. The $\eta$ parameter is defined such that the friction coefficient in Eq. (24) equals $\eta$. A straightforward generalizations^27^ shows that for interaction with local baths ${\sum }_{\alpha }\,{u}_{\alpha }({\boldsymbol{x}}){f}_{\alpha }(t)$, with ${u}_{\alpha }(x)=u(x-{x}_{\alpha })$, the effective friction coefficient is ${\eta }_{{\rm{eff}}}(x)=\eta \,{\sum }_{\alpha }\,{[{u{\prime} }_{\alpha }(x)]}^{2}$. For homogeneous distribution of local baths that have the same $\eta$, the friction coefficient becomes $x$-independent. In the model under consideration the coupling to the baths is ${\sum }_{\alpha }\,{{\boldsymbol{W}}}_{\alpha }\,{f}_{\alpha }(t)$, where $\alpha$ labels the sites. Disregarding commutation, it can be written as $2\,\cos ({\bf{p}})\,{\sum }_{\alpha }\,{u}_{\alpha }({\boldsymbol{x}}){f}_{\alpha }(t)$, where the $u$-s are site localized. It follow that the effective friction coefficient is momentum dependent, namely ${\eta }_{{\rm{eff}}}\sim |\,\cos (p{)|}^{2}\eta$. But for equilibration in the $\theta  < 1$ regime only low momenta are important hence we expect, up to numerical prefactor to observe ${\eta }_{{\rm{eff}}}\approx \eta$. The failure to observe this result is due to improper thermalization, as discussed in a previous paragraph.

### Positivity

Irrespective of $\eta$, there is another complication with the Ohmic master equation. If the temperature is low (small $\nu$) the relaxation may lead to a sub-minimal wavepacket that violates the uncertainty principle. This reflects the observation that the Ohmic master equation is not of Lindblad form, and violates the *positivity* requirement. The minimal correction required to restore positivity is to couple ***V*** to an extra noise source of intensity

28 $${\nu }_{\eta }=\frac{\nu }{{(4T)}^{2}}=\frac{{\eta }^{2}}{4\nu }$$

This term is essential in the low temperature regime. We have verified numerically that the extra noise term can be neglected in the high temperature regime where our interest is focused.

### On-site dissipators

The model of ^28^ combines Hamiltonian term with dissipator of the ${{\mathcal{L}}}^{({\rm{S}})}$ type that originates from couplings via ${{\boldsymbol{W}}}_{x}:\,={{\boldsymbol{Q}}}_{x}$, where ${{\boldsymbol{Q}}}_{x}=|x\rangle \langle x|$. Such dissipator leads to off-diagonal dephasing that is generated by ${{\boldsymbol{Q}}}_{x}\rho {{\boldsymbol{Q}}}_{x}$ terms, and therefore excludes the possibility for inter-site stochastic transitions. Similar remark applies to the familiar Caldeira-Leggett model of Quantum Brownian motion^25^, where the coupling is via ${\boldsymbol{W}}:\,={\boldsymbol{x}}$. Namely, it is a single bath that exerts a fluctuating homogeneous force that affects equally all the sites, as in^50^. In our model the dissipation effect is local: many uncorrelated local baths.

### Pauli dissipator

A conventional Pauli-type dissipator is obtained if we drop some of the terms in the Ohmic dissipator of Eq. (4). Namely,

29 $$\begin{array}{rcl}{{\mathcal{L}}}^{({\rm{Pauli}})}\rho  & = & -({w}^{+}+{w}^{-})\rho \\  &  & +\,\sum _{x}\,({w}^{+}{{\boldsymbol{D}}}_{x}\rho {{\boldsymbol{D}}}_{x}+{w}^{-}{{\boldsymbol{D}}}_{x}\rho {{\boldsymbol{D}}}_{x})\\  &  & -\,\gamma (\rho -\sum _{x}\,{{\boldsymbol{Q}}}_{x}\rho {{\boldsymbol{Q}}}_{x})\end{array}$$

The transition rates between sites, ${w}^{\pm }=[\nu \pm \eta {\mathcal{E}}]$, are in agreement with FGR. For completeness, we added here a $\gamma$ term that represents optional off-diagonal decoherence due to on-site noise.

### Ring configuration

For numerical treatment, and for the purpose of studying relaxation dynamics, we close the chain into a ring. This means to impose periodic boundary conditions. With uniform field ${\mathcal{E}}$, one encounter a huge potential drop at the boundary. To avoid this complication we assume that the boundary bond has an infinite temperature, hence the formation of a stochastic barrier is avoided, and the circulation of the stochastic field (aka *affinity*) becomes ${\mathcal{E}}/T$ as desired. In the analytical treatment of a clean ring we assume that $\nu$, and $\eta$ and ${\mathcal{E}}$ in the master equation are all uniform, such that invariance under translation is regained. This cheat is valid for large ring if $\rho$ is banded, reflecting a finite spatial correlation scale. See numerical verification in Supplementary S2.

### The Bloch eigenstates of a clean ring

The ${\mathcal{L}}$ super-operator in the Bloch $(r;q)$ basis, decomposes into $q$ Blocks. Each block can be written as a sum of terms, as in Eq. (9), that operate over a one dimensional tight binding $|r\rangle$ lattice. In this representation the coherent dynamics is generated by

30 $${{\mathcal{L}}}^{({\mathcal{E}})}=-\,i\,\sum _{r}\,|r\rangle r\langle r|$$

31 $${{\mathcal{L}}}^{(c)}=\,\sin (q\mathrm{/2)[}{{\mathcal{D}}}_{\perp }^{\dagger }-{{\mathcal{D}}}_{\perp }]$$

where ${{\mathcal{D}}}_{\perp }={\sum }_{r}\,|r+1\rangle \langle r|$. For the $\nu$ induced stochastic transitions we have

32 $${{\mathcal{L}}}^{(\nu )}=-\,2+2\,\cos (q)|0\rangle \langle 0|$$

33 $$\,+(|1\rangle \langle -\,1|+|-\,1\rangle \langle 1|)$$

where the last term is non-Pauli. The Pauli-type friction term takes the form:

34 $${{\mathcal{L}}}^{(\tilde{{\mathcal{E}}})}=-\,2i\,\sin (q)|0\rangle \langle 0|$$

And the additional friction terms are:

35 $${{\mathcal{L}}}^{(\tilde{c})}=\frac{1}{2}\,\cos (q/2)[{{\mathcal{D}}}_{\perp }+{{\mathcal{D}}}_{\perp }^{\dagger }]$$

36 $$\,+\,\frac{1}{2}\,\cos (3q/2)[|\pm \,1\rangle \langle 0|-|0\rangle \langle \pm \,1|]$$

37 $$\,+\,\frac{1}{2}\,\cos (q/2)[|\mp \,2\rangle \langle \pm \,1|-|\pm \,1\rangle \langle \mp \,2|]$$

Note that the zero eigenvalue belongs to the $q=0$ block. Some more details are provided in Supplementary S5.

### Diffusion at finite temperature

The Drude type term in the expression for the diffusion Eq. (20) is up to numerical prefactor $\langle {v}_{k}^{2}\rangle \tau$, where $\langle {v}_{k}^{2}\rangle ={c}^{2}/2$ for uniform momentum distribution. At finite temperature this distribution Eq. (15) is not uniform. Here we consider zero bias and get

38 $$\langle {v}_{k}^{2}\rangle ={\int }_{-\pi }^{\pi }\,{[c\sin (k)]}^{2}p(k)dk\approx \left[1-\frac{1}{8}{(\beta c)}^{2}\right]\frac{{c}^{2}}{2}$$

where $\beta =1/T$, and recall that $\eta =\nu /(2T)$. The analytical calculation in Supplementary S5 leads to a different result which implies that the expression in the square brackets should be replaced by $[1-6{\eta }^{2}]$, which means that the relevant dimensionless parameter is $\nu /T$ and not $c/T$. Figure Supplementary S4 of Supplementary S5 confirms this statement.

## Supplementary information

Supplementary Information.
