# Supplementary material for: Quantum stochastic transport along chains
Source: Sci Rep. 2020 Jun 25;10:10353. doi: 10.1038/s41598-020-66143-1 (PMC7316709; doi:10.1038/s41598-020-66143-1)
Supplement: Supplementary file 1 — Supplementary Information. [file 41598_2020_66143_MOESM1_ESM.pdf]

Quantum stochastic transport along chains

Dekel Shapira, Doron Cohen

(Supplementary Material)

[1] Ohmic dissipator for a two site system

Consider a two site system with Hamiltonian $\boldsymbol{H}_0$ and an Ohmic bath of temperature T that induces a fluctuating force $f(t)$ of intensity ν, and system-bath interaction term $-\boldsymbol{W}f(t)$. The master equation acquire a dissipation term

$$\mathcal{L}^{(\text{ohmic})}\rho \ = \ -\frac{\nu}{2}\,[\boldsymbol{W},[\boldsymbol{W},\rho]] - i\frac{\eta}{2}[\boldsymbol{W},\{\boldsymbol{V},\rho\}] \tag{S-1}$$

where $\boldsymbol{V} = i[\boldsymbol{H}_0,\boldsymbol{W}]$, and $\eta = \nu/(2T)$. An extra noise source $\tilde{f}(t)V$ can be added in order to make the right hand side "positive" in the Lindblad sense:

$$\mathcal{L}^{(\tilde{\nu})}\rho \ = \ -\frac{\nu_\eta}{2}\,[\boldsymbol{V},[\boldsymbol{V},\rho]] \tag{S-2}$$

The "minimal correction" that is needed is to set $\nu_\eta = \nu/(4T)^2$, and then the expression can be written in the Lindblad form

$$\mathcal{L}^{(\text{ohmic})}\rho \ = \ \nu\left(\boldsymbol{F}\rho\boldsymbol{F}^\dagger - \frac{1}{2}\left\{\boldsymbol{F}^\dagger\boldsymbol{F},\rho\right\}\right) - i[\boldsymbol{H}_{LS},\rho] \tag{S-3}$$

$$\boldsymbol{F} \ = \ \boldsymbol{W} + i\frac{\eta}{2\nu}\boldsymbol{V} \tag{S-4}$$

$$\boldsymbol{H}_{LS} \ = \ \frac{\eta}{4}[\boldsymbol{W}\boldsymbol{V}+\boldsymbol{V}\boldsymbol{W}] \tag{S-5}$$

where the Lamb-shift term $\boldsymbol{H}_{LS}$ can be absorbed into the system Hamiltonian. For two site system with

$$\boldsymbol{H}_0 \ = \ -(\mathcal{E}/2)\boldsymbol{\sigma}^z - (c/2)\boldsymbol{\sigma}^x \tag{S-6}$$

and coupling $\boldsymbol{W} = \boldsymbol{\sigma_x}/2$, one has $\boldsymbol{V} = \mathcal{E}\boldsymbol{\sigma_y}$, and the Lamb-shift is zero. The Lindblad generator is

$$\boldsymbol{F} \ = \ \left(1+\frac{\mathcal{E}}{T}\right)\boldsymbol{\sigma}^+ + \left(1-\frac{\mathcal{E}}{T}\right)\boldsymbol{\sigma}^- \tag{S-7}$$

The transition rates between the sites are:

$$w^\pm \ = \ \left(1\pm\frac{\eta\mathcal{E}}{2\nu}\right)^2\nu \ \approx \ (\nu\pm\eta\mathcal{E}) \tag{S-8}$$

$$\frac{w^-}{w^+} \ \approx \ e^{-\mathcal{E}/T} \tag{S-9}$$

A secular-like (Pauli) version of the dissipator is obtained by expanding $\boldsymbol{F}\rho\boldsymbol{F}^\dagger$ and keeping only the Lindblad terms with $\boldsymbol{F}_+ = \boldsymbol{\sigma}^+$ and $\boldsymbol{F}_- = \boldsymbol{\sigma}^-$. Namely,

$$\mathcal{L}^{(\text{Pauli})}\rho = w^+\left(\boldsymbol{\sigma}^+\rho\boldsymbol{\sigma}^- - \frac{1}{2}\{\boldsymbol{\sigma}^-\boldsymbol{\sigma}^+,\rho\}\right) + w^-\left(\boldsymbol{\sigma}^-\rho\boldsymbol{\sigma}^+ - \frac{1}{2}\{\boldsymbol{\sigma}^+\boldsymbol{\sigma}^-,\rho\}\right) - \frac{\gamma}{4}\,[\boldsymbol{\sigma}^z,[\boldsymbol{\sigma}^z,\rho]] \tag{S-10}$$

where the last term represents excess noise due to noisy detuning (see below). The mixed terms that have been omitted affect only the decoherence of the off-diagonal terms, and not the rate of transitions between sites. In the Bloch-vector representation the precessing component of the "spin" decays only in the y direction in the Ohmic version, and isotropically in the Pauli version. In some sense the dissipation in the Pauli version assumes two independent baths at each bond. If we assume that the detuning $\mathcal{E}$ is fluctuating with intensity $\nu_\gamma = (\gamma/2)$, so that $(\mathcal{E}/2) \to (\mathcal{E}/2) + f(t)$, then an additional $\mathcal{L}$ term appears, that has the form of Eq.(S-1) with the substitution $\boldsymbol{W} := \boldsymbol{\sigma}^z$, and $\boldsymbol{V} := -c\boldsymbol{\sigma}^y$.

[2] Ohmic dissipator for a chain

The Hamiltonian of the chain is

$$\boldsymbol{H}^{(c)} \;=\; U(\boldsymbol{x}) - \frac{c}{2}(\boldsymbol{D}+\boldsymbol{D}^{\dagger}) \;=\; -\mathcal{E}\boldsymbol{x} - c\cos(\boldsymbol{p}) \tag{S-11}$$

where $\boldsymbol{D} = \sum_x \boldsymbol{D}_x$ is the displacement operator, and $\boldsymbol{D}_x = |x+1\rangle\langle x|$. In general the field $\mathcal{E}_x = -\left(U(x{+}1) - U(x)\right)$, as well as the hopping frequencies (c), and temperatures might be non-uniform. The interaction with a bath-source that induces non-coherent transitions at a given bond is obtained by the replacement $(c/2) \mapsto (c/2) + f(t)$. The baths of different bonds are uncorrelated. Accordingly the dissipation term in the Master equation takes the form

$$\mathcal{L}^{(\text{ohmic})}\rho = -\sum_x \left(\frac{\nu}{2}[\boldsymbol{W}_x,[\boldsymbol{W}_x,\rho]] + \frac{\eta}{2}\, i[\boldsymbol{W}_x,\{\boldsymbol{V}_x,\rho\}]\right) \tag{S-12}$$

where the coupling to the baths is via the operators

$$\boldsymbol{W}_x = \left(\boldsymbol{D}_x + \boldsymbol{D}_x^{\dagger}\right) \tag{S-13}$$

$$\boldsymbol{V}_x = i[\boldsymbol{H}^{(c)},\boldsymbol{W}_x] = i\mathcal{E}_x\left(\boldsymbol{D}_x^{\dagger} - \boldsymbol{D}_x\right) - i\frac{c}{2}\left[(\boldsymbol{D}_{x+1}\boldsymbol{D}_x - \boldsymbol{D}_x\boldsymbol{D}_{x-1}) - h.c\right] \tag{S-14}$$

And the Lindblad correction term:

$$\mathcal{L}^{(\tilde{\nu})}\rho \;=\; -\frac{\nu_\eta}{2}\sum_x[\boldsymbol{V}_x,[\boldsymbol{V}_x,\rho]] \tag{S-15}$$

with intensity $\nu_\eta = \nu/(4T)^2$. Such term has negligible effect in the high temperature regime ($\eta < 1$). Optionally we can add terms that reflect fluctuations of the field. At a given bond it is obtained by the replacement $U(\boldsymbol{x}) \mapsto U(\boldsymbol{x}) + \tilde{f}(t)$, where $\tilde{f}(t)$ represents fluctuations of intensity γ. The implied coupling operators are

$$\boldsymbol{W}_x^{(S)} \;=\; \boldsymbol{Q}_x \tag{S-16}$$

$$\boldsymbol{V}_x^{(S)} \;=\; i[\boldsymbol{H}^{(c)},\boldsymbol{W}_x^{(D)}] \;=\; i(c/2)\left[\boldsymbol{D}_{x-1}^{\dagger} - \boldsymbol{D}_{x-1} - \left(\boldsymbol{D}_x^{\dagger} - \boldsymbol{D}_x\right)\right] \tag{S-17}$$

[3] Expression for the current

For generality of the treatment we allow the temperature to be bond dependent, then $\eta \to \eta_x$ so that the Lindblad generators are $\boldsymbol{F}_x = \boldsymbol{W}_x + i(\eta_x/2\nu)\boldsymbol{V}_x$, and

$$\boldsymbol{H} = \boldsymbol{H}^{(c)} + \sum_x \frac{\eta_x}{4}\{\boldsymbol{W}_x,\boldsymbol{V}_x\} \tag{S-18}$$

The time dependence of an expectation value is given by the adjoint equation:

$$\frac{d}{dt}\langle\boldsymbol{Q}\rangle \;=\; \text{trace}\left[\boldsymbol{Q}\frac{d}{dt}\rho\right] \;=\; \text{trace}\left[\boldsymbol{Q}\mathcal{L}\rho\right] \;=\; \text{trace}\left[(\mathcal{L}^{\dagger}\boldsymbol{Q})\rho\right] \;=\; \left\langle\mathcal{L}^{\dagger}\boldsymbol{Q}\right\rangle \tag{S-19}$$

where

$$\mathcal{L}^{\dagger}\boldsymbol{Q} \;=\; i[\boldsymbol{H},\boldsymbol{Q}] + \nu\sum_x\left(\boldsymbol{F}_{\boldsymbol{x}}^{\dagger}\boldsymbol{Q}\boldsymbol{F}_{\boldsymbol{x}} - \frac{1}{2}\{\boldsymbol{F}_{\boldsymbol{x}}^{\dagger}\boldsymbol{F}_{\boldsymbol{x}},\boldsymbol{Q}\}\right) \tag{S-20}$$

Partitioning the system at the n-th bond, the current flowing from left to right is defined by $I = \langle\dot{\boldsymbol{Q}}\rangle$, with

$$\boldsymbol{Q} \;=\; \sum_{x>n}|x\rangle\langle x| \tag{S-21}$$

We note that although the original Hamiltonian allows only near-neighbor hopping, the master equation allows also "double hopping" due to the $\boldsymbol{V}$ terms. Accordingly the expression for the current operator has several non-trivial

terms. Applying Eq.(S-19) the current is:

$$I = \vec{I} - \overleftarrow{I} - c\,\mathrm{Im}[\rho_n(1)] + I^{(0)}_{\eta^2} + I^{(1)}_{\eta^2} \tag{S-22}$$

$$\vec{I} = w_n^+ p_n - \frac{c\eta_n}{2}\mathrm{Re}[\rho_{n-1}(1)] \tag{S-23}$$

$$\overleftarrow{I} = w_n^- p_{n+1} - \frac{c\eta_n}{2}\mathrm{Re}[\rho_{n+1}(1)] \tag{S-24}$$

$$I^{(0)}_{\eta^2} = \frac{\mathcal{E}^2\eta_n^2}{4\nu}[p_n - p_{n+1}] + \sum_{i=0,1}\frac{c^2}{16\nu}\left(\eta^2_{n-i} + \eta^2_{n+1-i}\right)\left(p_{n-i} - p_{n+2-i}\right) \tag{S-25}$$

$$I^{(1)}_{\eta^2} = -\frac{c\mathcal{E}}{8\nu}\mathrm{Re}\left[2\eta_n^2\left(\rho_{n-1}(1) + \rho_{n+1}(1)\right) - \left(\eta^2_{n-1} + \eta^2_{n+1}\right)\rho_n(1)\right] \tag{S-26}$$

where the extra I_{η^2} terms are of order η^2, and are negligible for the NESS current. If the field $\mathcal{E}$ is non-uniform, then one needs to make the replacement $\eta_n\mathcal{E} \to \eta_n\mathcal{E}_n$. Disregarding I_{η^2} the distinct elements of the current are coherent hopping, stochastic hopping and stochastic-assisted coherent hopping. These are pictured in Fig.S1.

Current in disordered system.– In Fig.S2 we display results for the NESS current, calculated for a disordered sample. If the spatial correlation scale of the disorder is large, the ring can be regarded as composed of several segments connected in series. Then the analytical estimate for the current would be

$$I = \left[\sum_x \frac{1}{v(\mathcal{E}_x)}\right]^{-1} \tag{S-27}$$

which reduce to $I = (1/L)v(\mathcal{E})$ for a uniform field. The function $v(\mathcal{E})$ is provided by Eq.(17), and the above analytical estimate implies what we call *convex property*. Using this formula we can explain why disorder can lead to increase of the current as in Fig.2. The accuracy of this formula, that assumes a large spatial correlation scale, is tested against the correlation scale in Fig.S2.

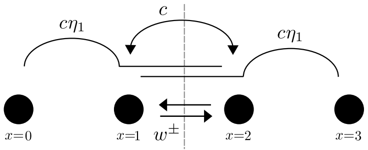

FIG. S1. Diagrammatic representation of the terms that contributes to the total current that flows via a section that is indicated by a vertical dashed line, here via the bond that connects sites x=1 and x=2. Straight lines denotes the role played by the stochastic transitions, while semi-circle segments are related to coherent hopping. The latter are of the form $c\eta_x\rho_x(1)$.

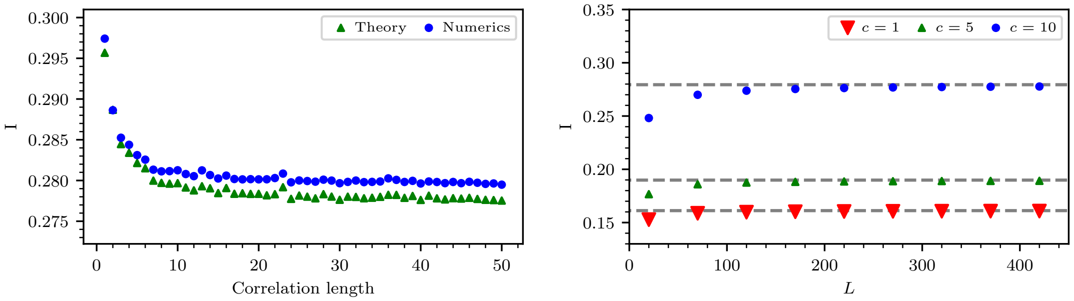

FIG. S2. The NESS current as a function of the correlation length of the disorder. The parameters are $\mathcal{E}$=8, $\sigma_{\mathcal{E}}$=6, η=0.01 and c=10. The correlated disorder is obtained by a convolution of the uncorrelated disorder (correlation length equals unity) with a box-shaped kernel. For each sample the numerical result for an L=500 ring is compared with the theoretical estimate Eq.(S-27). The two are expected to coincide for large correlation length. The small difference that remains is a finite size effect due to our treatment of the boundary conditions (see main text). On the right panel we demonstrate the L-dependence of this difference for a clean system ($\mathcal{E}$ and η are the same). The gray dashed lines are the theoretical values provided by Eq.(17).

[4] Spreading

Without coherent hopping (c=0) the Ohmic/Pauli dynamics of the on-site probabilities p_x decouple from the decay of the off-diagonal terms. We get two distinct sets of modes: the stochastic-like relaxation modes and the off-diagonal decoherence modes. In the Pauli approximation the latter decay with the *same* rate $\gamma_0 = \gamma + w^+ + w^-$. The stochastic transitions affect only the stochastic-like relaxation modes. Starting with a wavepacket of variance $\text{Var}(R) = \sigma_0^2$, and momentum centered around k_0, we get in the Wigner representation

$$\rho_w(R,P) \;=\; e^{-\gamma_0 t}\left[G^c(R,P) - G^0(R,P)\right] \;+\; G^t(R,P) \tag{S-28}$$

where

$$G^c(R,P) \;=\; \frac{2}{L}\exp\left(-\frac{1}{2}\frac{R^2}{\sigma_0^2} - (P-k_0(t))^2\sigma^2\right), \qquad k_0(t) = k_0 + \mathcal{E}t \tag{S-29}$$

$$G^t(R,P) \;=\; \frac{1}{\sqrt{2\pi}}\frac{1}{\sqrt{\sigma_0^2+2Dt}}\exp\left(-\frac{1}{2}\frac{(R-vt)^2}{\sigma_0^2+2Dt}\right) \tag{S-30}$$

with drift velocity $v = (w^+ - w^-)$ and diffusion coefficient $D = (w^+ + w^-)/2$.

Let us add coherent hopping in a very naive way: we use the Pauli dissipator, and merely set $c \neq 0$ in the Hamiltonian. It is a naive procedure because the use of the Pauli dissipator cannot be justified anymore (we have to use the Ohmic dissipator). With this simplification the explicit form of the Lindblad operator of the q block is:

$$\mathcal{L}^{(q)} \;=\; -\gamma_0 \;+\gamma_q|0\rangle\langle 0| \;-i\mathcal{E}\sum_r |r\rangle\, r\,\langle r| \;-c\sin(q/2)\left[e^{iq/2}\mathcal{D}_\perp - e^{-iq/2}\mathcal{D}_\perp^\dagger\right] \tag{S-31}$$

with $\gamma_q = \gamma + w^+e^{-iq} + w^-e^{iq}$. This can be regarded as simplified version of Eq.(S-33) below. The diagonalization of $\mathcal{L}$ is straightforward for zero bias. The phases $\exp(\pm iq/2)$ can be gauged to some distant r, and $\mathcal{L}$ becomes like the Hamiltonian of a tight-binding model with a barrier at the origin. The lowest eigenmodes are decaying exponents $\psi(r) \sim \exp(-\alpha|r|)$, with $\text{Re}(\alpha) > 0$. These modes correspond to the stochastic-like relaxation modes. Matching the boundary conditions at $r = 0$, one finds the eigenvalues

$$\lambda_{q,0} \;=\; \gamma_0 - \sqrt{\gamma_q^2 - 4c^2\sin^2(q/2)} \;\equiv\; ivq + Dq^2 + O(q^3) \tag{S-32}$$

From which expressions for v and D can be derived. The result for D is similar (but not identical) to the correct result in the main text. Namely, up to a prefactor it reproduces the $\mathcal{E}$=0 Drude term.

[5] Bloch representation of the Ohmic master equation

For a clean system, and neglecting the η^2 contribution, the generator of the master equation is written as a sum of several terms. Here we shall provide explicit expressions of the q block of the super-matrix in the Bloch representation:

$$\mathcal{L}^{(q)} \;=\; c\mathcal{L}^{(c)} + \mathcal{E}\mathcal{L}^{(\mathcal{E})} + \nu\mathcal{L}^{(\nu)} + \eta c\mathcal{L}^{(\tilde{c})} + \eta\mathcal{E}\mathcal{L}^{(\tilde{\mathcal{E}})} + \nu_\eta\mathcal{L}^{(\tilde{\nu})} \tag{S-33}$$

We define operators

$$R \;=\; \sum_r |r\rangle\, r\,\langle r| \tag{S-34}$$

$$\mathcal{D}_\perp \;=\; \sum_r |r+1\rangle\langle r| \tag{S-35}$$

After gauge transformation $|r\rangle \to e^{-iqr/2}|r\rangle$ we obtain

$$\mathcal{L}^{(c)} \;=\; \sin(q/2)[\mathcal{D}_\perp^\dagger - \mathcal{D}_\perp] \tag{S-36}$$

$$\mathcal{L}^{(\mathcal{E})} \;=\; -iR \tag{S-37}$$

$$\mathcal{L}^{(\nu)} \;=\; -2 + 2\cos(q)|0\rangle\langle 0| + (|1\rangle\langle -1| + |-1\rangle\langle 1|) \tag{S-38}$$

$$\mathcal{L}^{(\tilde{c})} \;=\; \frac{1}{2}\cos(q/2)[\mathcal{D}_\perp + \mathcal{D}_\perp^\dagger] + \frac{1}{2}\cos(3q/2)\,[|\pm 1\rangle\langle 0| - |0\rangle\langle \pm 1|] + \frac{1}{2}\cos(q/2)\,[|\mp 2\rangle\langle \pm 1| - |\pm 1\rangle\langle \mp 2|] \tag{S-39}$$

$$\mathcal{L}^{(\tilde{\mathcal{E}})} \;=\; -2i\sin(q)|0\rangle\langle 0| \tag{S-40}$$

Note that this expression is not 2π periodic, since we ignore the accumulated phase which arise in the gauge procedure. The gauge in the above procedure is equivalent to redefinition of the r coordinate such that x and r become orthogonal (skewing the r axis in Fig.4 by 45 degrees).

[6] Eigenmodes of the Ohmic master equation

Infinite temperature eigen-modes.– For infinite temperature ($\eta = 0$), the eigenvalues of the $q = 0$ block are:

$$\lambda_{q=0,0} = 0 \text{ (NESS)} \tag{S-41}$$
$$\lambda_{q=0,\pm} = 2\nu \pm \sqrt{\nu^2 - \mathcal{E}^2} \tag{S-42}$$
$$\lambda_{q=0,s} = 2\nu + i\mathcal{E}s, \quad (s = \pm 2, \pm 3, ...) \tag{S-43}$$

Considering the q dependence of the eigenvalues we get several bands. Our interest below is in the lowest band ($\lambda_{q,s=0}$), which determines the long time spreading. For this calculation one needs the eigen-modes corresponding to the above eigenvalues. These are given by:

$$|\lambda_{q=0,s}\rangle = |r = s\rangle, \quad (s = 0, \pm 2, \pm 3, ...) \tag{S-44}$$
$$|\lambda_{q=0,\pm}\rangle \equiv |\pm\rangle = \alpha_\pm |1\rangle + |-1\rangle \quad \text{(unnormalized)} \tag{S-45}$$
$$\alpha_\pm = -i\left(\frac{\mathcal{E}}{\nu}\right) \mp \sqrt{1 - \left(\frac{\mathcal{E}}{\nu}\right)^2} \tag{S-46}$$

NESS at finite temperature.– We can find the NESS, which is the zero mode $|\lambda_{0,0} = 0\rangle$, and calculate from it both the momentum distribution and the current.

Setting $q = 0$, and considering linear order in η, the NESS is obtained by first order perturbation for the $|\lambda_{q=0,0}\rangle = |r = 0\rangle$ state. See Fig.S3. Putting $V \equiv \eta c \mathcal{L}^{(\bar{c})}$ as the perturbation, one get:

$$|\text{NESS}\rangle = |0\rangle + \frac{\langle\tilde{+}|V|0\rangle}{\lambda_+}|+\rangle + \frac{\langle\tilde{-}|V|0\rangle}{\lambda_-}|-\rangle = |0\rangle + \alpha_0|1\rangle + \alpha_0^*|-1\rangle \tag{S-47}$$
$$\alpha_0 = \frac{3\nu - i\mathcal{E}}{3\nu^2 + \mathcal{E}^2}\eta c \tag{S-48}$$

where the left eigenvectors are given by:

$$\langle\tilde{\pm}| = \left(\frac{\alpha_\pm}{\alpha_\mp} - 1\right)^{-1}\left[\langle 1|\frac{1}{\alpha_\mp} - \langle -1|\right], \tag{S-49}$$

Reverting back from the Bloch basis of $\rho(r;q)$ to the position basis, namely $|r;q\rangle := L^{-\frac{1}{2}}\sum_x |x\rangle\langle x+r|e^{iqx}$, the normalized steady state matrix ρ is:

$$\rho^{(\text{NESS})} = \frac{1}{L}\left(\mathbb{1} + \sum_x \alpha_0|x\rangle\langle x+1| + h.c.\right) = \frac{1}{L}\left(\mathbb{1} + \alpha_0 e^{+ip} + \alpha_0^* e^{-ip}\right) \tag{S-50}$$

The momentum distribution.– Using Eq.(S-50) we obtain the steady state momentum distribution:

$$p(k) = \rho_{kk} = \frac{1}{L}\left(1 + 2\text{Re}(\alpha_0 e^{+ik})\right) = \frac{1}{L} + \frac{1}{L}\frac{2\eta c}{3\nu^2 + \mathcal{E}^2}\left(3\nu\cos(k) + \mathcal{E}\sin(k)\right) \tag{S-51}$$

For $\mathcal{E} = 0$, the momentum distribution is canonical, see Fig. S4. The above result is indeed consistent with the canonical distribution to linear order in $\beta = 1/T$. The drift velocity can be deduced by calculating the NESS current using Eq.(S-22). The current over the bond n, to first order in η is:

$$I_n = \frac{1}{L}\left((w_n^+ - w_n^-) - c\,\text{Im}(\alpha_0)\right) = \frac{1}{L}\left[1 + \frac{c^2}{6\nu^2 + 2\mathcal{E}^2}\right]2\eta\mathcal{E} \tag{S-52}$$

We note that although the expression for the current Eq.(S-22) is complicated, the final NESS current is composed of the usual stochastic-current, and the usual coherent-current.

[7] Diffusion at finite temperature

Here we provide the calculation of D to second order in η. We have to expand $\lambda_{q,0}$ to second order in q. Inspecting the gauged Lindblad operator in Eq.(S-33), one observes (see diagram of Fig.S3) that up to order q^2 and η^2 it is enough to diagonalize the five sites $|r| \le 2$, keeping the q^2 and the η^2 corrections. This can be done using perturbation theory, or optionally using *Mathematica* for a direct diagonlization and then expand the result in powers of q and η. Either way one get:

$$\lambda_{q,0} = ivq + Dq^2 \tag{S-53}$$

$$v = \left[1 + \frac{c^2}{6\nu^2 + 2\mathcal{E}^2}\right] 2\eta\mathcal{E} \tag{S-54}$$

$$D = \left[1 + \frac{c^2}{6\nu^2 + 2\mathcal{E}^2}\right]\nu - \left[\frac{\left(9\nu^2 + 11\mathcal{E}^2\right)}{(\mathcal{E}^2 + 3\nu^2)^2} + \frac{\left(15\mathcal{E}^2 + 13\nu^2\right)(c\mathcal{E})^2}{4(\mathcal{E}^2 + \nu^2)(\mathcal{E}^2 + 3\nu^2)^3}\right](\eta c)^2\nu \tag{S-55}$$

Setting $\mathcal{E} = 0$ in the expression for D, we find that the η^2 correction in Eq.(S-55) can be absorbed into the first term via the replacement $c^2 \mapsto [1 - 6\eta^2]c^2$. Note that this correction is based on the Ohmic dissipator without the additional Lindblad term that is added for the purpose of positivity.

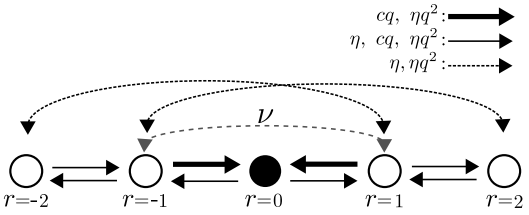

FIG. S3. Diagrammatic representation of the couplings in the reduced tight binding model (in r), that is used in order to determine the eigenvalues $\lambda_{q,s}$ for a given Bloch momentum q. Different orders of q and η are indicated by the different arrows. The formation of the $|\lambda_{q=0,\pm}\rangle$ eigenmodes is due to the dashed ν coupling. Up to order q^2 and η it is enough to consider second order perturbation theory involving $r = -1, 0, 1$. For η^2 corrections one needs to include also $r = \pm 2$.

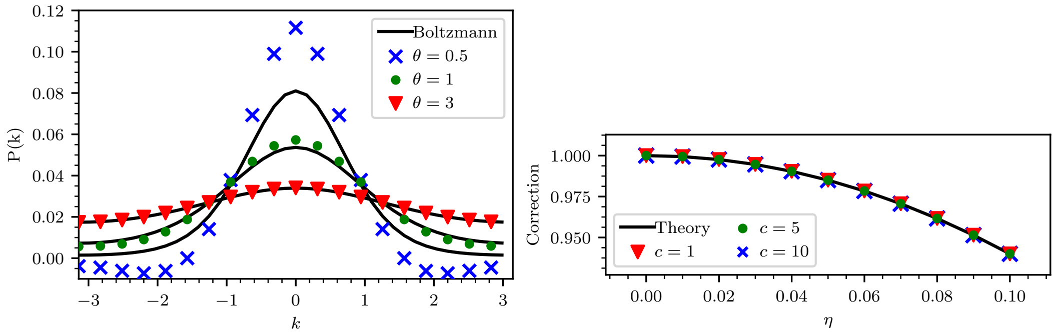

FIG. S4. The steady state and the diffusion coefficient at zero bias. The parameters are $\nu = 1$ and $\eta = 0.01$. **(a)** Numerically determined momentum distribution (without the Lindblad correction) compared with the Boltzmann distribution $p(k) \propto \exp\left[\theta^{-1}\cos(k)\right]$. The distribution is plotted for a few values of θ. For small θ the distribution is no longer Boltzmann-like and its tails become negative. The Ohmic master equation is no longer valid in this regime. The calculation is for $L = 40$, and for clarity only 20 data points are presented. **(b)** Numerical verification of the η^2 correction to D. The analytically predicted correction factor $[1 - 6\eta^2]$ is plotted as a solid line. The numerical data points are $[6\nu/c^2](D - \nu)$.

[8] Effective disorder

For c=0 the off-diagonal terms of ρ decouple from the diagonal, and the relaxation spectrum is the same as that of the stochastic model. For small c, we keep only the three-central diagonals ($r \leq 1$), thus ignoring the couplings to higher bands. We also ignore transitions of the order $c\eta$. The $r = \pm 1$ space can be eliminated, in the price of getting a λ-dependent transition rate matrix $H_{\text{eff}}(\lambda) = H_0 + W'G(\lambda)W$, where H_0 includes the transitions along r=0, and $G(\lambda)$ is a resolvent operator that describes the dynamics within the excluded diagonal, while the W-s include the couplings between the r=0 elements and the excluded $r = \pm 1$ elements. See diagram of the couplings in Fig.S5. The effective matrix $H_{\text{eff}}(\lambda)$ is non-hermitian due to the asymmetry of the transitions. It is a probability conserving tight-binding operator, but with rates that can be negative. For the forward and backward hopping rates in the n-th bond we get $w_n^{\pm} = \nu + \nu_n \pm \eta\mathcal{E}_n$, where

$$\nu_n \;=\; \left(\frac{c}{2}\right)^2 (G_{11} + G_{22} - G_{12} - G_{21}) \;=\; \frac{c^2}{2} \frac{\nu - \lambda}{(2\nu - \lambda)^2 + \mathcal{E}_n^2 - \nu^2} \tag{S-56}$$

where $G = -(\lambda + L_n)^{-1}$ is a 2×2 matrix which is defined in terms of $L_n = -2\nu - i\mathcal{E}_n\boldsymbol{\sigma}_z + \nu\boldsymbol{\sigma}_x$ within the subspace that is spanned by the super-vectors $|n\rangle\langle n+1|$ and $|n+1\rangle\langle n|$. The eigenvectors of this matrix are the $|\pm\rangle$ of Eq.(S-46) with $\mathcal{E} \to \mathcal{E}_n$.

In order to estimate the effective disorder, we proceed as outlined in the main text. For high-temperatures one obtains from Eq. (22) approximations for the w_n and for the stochastic field, namely, $w_n \approx (\nu + \nu_n)$, and $\tilde{\mathcal{E}}_n \approx \eta\mathcal{E}(\nu + \nu_n)^{-1}$. We define an associated hermitian matrix $\tilde{H}$, that has the same matrix elements as $H_{\text{eff}}(\lambda)$, but with $\tilde{\mathcal{E}}_n = 0$ in the off diagonal elements. The eigenvalues of $\tilde{H}$ are real, with some inverse localization length $\kappa(\lambda)$. Ignoring the diagonal disorder that arises due to non-uniform field $\tilde{\mathcal{E}}_n$, the localization length of eigenvalues near λ are roughly given by [Weinberg, de Leeuw, Kottos, Cohen, Phys. Rev. E 93, 062138 (2016)]:

$$\kappa(\lambda) \;\approx\; \frac{1}{4}\left(\frac{\sigma_\perp}{\nu}\right)^2 \frac{\lambda}{\nu} \tag{S-57}$$

with $\sigma_\perp^2 = \text{Var}(w_n)$. This, as explained in the main text, determines whether the eigenvalues of $H_{\text{eff}}(\lambda)$ will turn complex. We focus on representative region around $\lambda = 2\nu$ in the center of the spectrum. Around this point, for small disorder, one obtains:

$$w_n \;\approx\; \nu\frac{c^2}{2(\nu^2 - \mathcal{E}^2)}\left(1 + B\delta_n + C\delta_n^2\right) \tag{S-58}$$

$$B = \frac{2\mathcal{E}}{(\nu^2 - \mathcal{E}^2)}, \qquad C = \frac{\nu^2 + 3\mathcal{E}^2}{(\nu^2 - \mathcal{E}^2)^2} \tag{S-59}$$

with $\delta_n \equiv \mathcal{E}_n - \mathcal{E}$ that are randomly distributed within $[-\sigma_{\mathcal{E}}, \sigma_{\mathcal{E}}]$. Consequently we get the estimate

$$\sigma_\perp^2 \;=\; \text{Var}(w_n) \;\approx\; \left(\frac{c^2\nu}{2(\nu^2 - \mathcal{E}^2)}\right)^2 \left(B^2\,\text{Var}(\delta) + C^2\,\text{Var}(\delta^2)\right) = \left(\frac{c^2\nu}{2(\nu^2 - \mathcal{E}^2)}\right)^2 \left(B^2(\sigma^2/3) + C^2(4\sigma^4/45)\right)$$

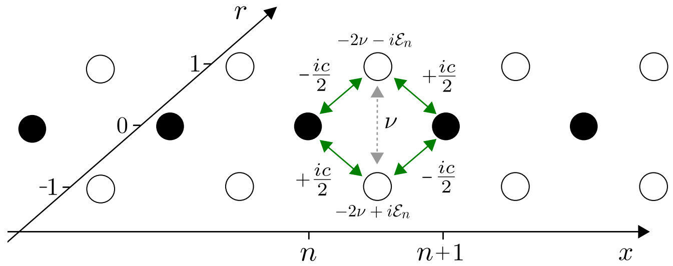

FIG. S5. Diagrammatic representation of the couplings in the 3 band approximation. Along the main diagonal (filled circles) we have asymmetric stochastic transitions (not indicated). Those are coupled to the coherences (empty circles) due to the c-related terms that are packed into W and W' matrices. The non-Pauli ν coupling and the on-site "energies" at the $|r| = 1$ sites constitute the L_n operator, which determines the resolvent $G(\lambda)$.

Acknowledgment.– This research was supported by the Israel Science Foundation (Grant No.283/18).

[R1] Optimal transport in one-dimensional infinite chains has been studied in [5–7, 9, 18, 19].
[R2] Optimal transport in one-dimensional chains with "exit site" has been studied in [12–14]
[R3] The term quantum Goldilocks effect has been suggested in [10], for the idea that natural selection tends to drive quantum systems to the degree of optimal quantum coherence for transport.
[S1] See SM sections 1 and 2 for pedagogical presentation of the procedure for the construction of an Ohmic master equation for 2 a site system and for a chain.
[S2] See SM section 3 for an explicit expression for the current operator.
[S3] See SM section 4 for pedagogical summary regarding spreading, following [27].
[S4] See SM section 4 for technical summary of the procedure for finding the eigenvalues of a master equation with a Pauli-type dissipator. It follows [28], but here we include additional stochastic transitions in "parallel" to the coherent hopping, and incorporate also the bias within a first order treatment.
[S5] See SM sections 5-7 for technical details regarding the procedure for finding the eigenmodes of the Ohmic master equation, including explicit expressions for the η related terms in the Fourier representation, numerical verification for momentum thermalization, and derivation of the associated η-related correction for the diffusion coefficient.
[S6] See SM section 3 for extra numerics that concerns the calculation of the current for a disordered chain, and the manifestation of the convex property.
[S7] See SM section 8 for technical details regarding the derivation of the effective disorder that emerges in the reduced rate equation due to virtual coherent transitions.

[1] T Hartmann, F Keck, H J Korsch, and S Mossmann, "Dynamics of bloch oscillations," New Journal of Physics **6**, 2–2 (2004).
[2] Francois Dubin, Romain Melet, Thierry Barisien, Roger Grousson, Laurent Legrand, Michel Schott, and Valia Voliotis, "Macroscopic coherence of a single exciton state in an organic quantum wire," Nature Physics **2**, 32 (2006).
[3] Tammie R Nelson, Dianelys Ondarse-Alvarez, Nicolas Oldani, Beatriz Rodriguez-Hernandez, Laura Alfonso-Hernandez, Johan F Galindo, Valeria D Kleiman, Sebastian Fernandez-Alberti, Adrian E Roitberg, and Sergei Tretiak, "Coherent exciton-vibrational dynamics and energy transfer in conjugated organics," Nature communications **9**, 2316 (2018).
[4] Thomas Dekorsy, Albrecht Bartels, Heinrich Kurz, Klaus Köhler, Rudolf Hey, and Klaus Ploog, "Coupled bloch-phonon oscillations in semiconductor superlattices," Physical review letters **85**, 1080 (2000).
[5] A Madhukar and W Post, "Exact solution for the diffusion of a particle in a medium with site diagonal and off-diagonal dynamic disorder," Physical Review Letters **39**, 1424 (1977).
[6] Ulrich Weiss and Hermann Grabert, "Quantum diffusion of a particle in a periodic potential with ohmic dissipation," Physics Letters A **108**, 63–67 (1985).
[7] N. Kumar and A. M. Jayannavar, "Quantum diffusion in thin disordered wires," Phys. Rev. B **32**, 3345–3347 (1985).
[8] Dibyendu Roy, "Crossover from ballistic to diffusive thermal transport in quantum langevin dynamics study of a harmonic chain connected to self-consistent reservoirs," Physical review. E, Statistical, nonlinear, and soft matter physics **77**, 062102 (2008).
[9] Ariel Amir, Yoav Lahini, and Hagai B Perets, "Classical diffusion of a quantum particle in a noisy environment," Physical Review E **79**, 050105 (2009).
[10] Seth Lloyd, Masoud Mohseni, Alireza Shabani, and Herschel Rabitz, "The quantum goldilocks effect: on the convergence of timescales in quantum transport," arXiv:1111.4982 (2011).
[11] Jeremy M Moix, Michael Khasin, and Jianshu Cao, "Coherent quantum transport in disordered systems: I. the influence of dephasing on the transport properties and absorption spectra on one-dimensional systems," New Journal of Physics **15**, 085010 (2013).
[12] Jianlan Wu, Robert J. Silbey, and Jianshu Cao, "Generic mechanism of optimal energy transfer efficiency: A scaling theory of the mean first-passage time in exciton systems," Phys. Rev. Lett. **110**, 200402 (2013).
[13] Yang Zhang, G. Luca Celardo, Fausto Borgonovi, and Lev Kaplan, "Opening-assisted coherent transport in the semiclassical regime," Phys. Rev. E **95**, 022122 (2017).
[14] Yang Zhang, G. Luca Celardo, Fausto Borgonovi, and Lev Kaplan, "Optimal dephasing for ballistic energy transfer in disordered linear chains," Phys. Rev. E **96**, 052103 (2017).
[15] Herbert van Amerongen, Rienk van Grondelle, and Leonas Valkunas, *Photosynthetic Excitons* (WORLD SCIENTIFIC, 2000).
[16] Thorsten Ritz, Ana Damjanović, and Klaus Schulten, "The quantum physics of photosynthesis," ChemPhysChem **3**, 243–248 (2002).
[17] Yuan-Chung Cheng and Graham R. Fleming, "Dynamics of light harvesting in photosynthesis," Annual Review of Physical Chemistry **60**, 241–262 (2009).
[18] Martin B Plenio and Susana F Huelga, "Dephasing-assisted transport: quantum networks and biomolecules," New Journal of Physics **10**, 113019 (2008).
[19] Patrick Rebentrost, Masoud Mohseni, Ivan Kassal, Seth Lloyd, and Alán Aspuru-Guzik, "Environment-assisted quantum

transport," New Journal of Physics **11**, 033003 (2009).
[20] Patrick Rebentrost, Masoud Mohseni, and Aln Aspuru-Guzik, "Role of quantum coherence and environmental fluctuations in chromophoric energy transport," The Journal of Physical Chemistry B **113**, 9942–9947 (2009).
[21] Mohan Sarovar and K Birgitta Whaley, "Design principles and fundamental trade-offs in biomimetic light harvesting," New Journal of Physics **15**, 013030 (2013).
[22] KDB Higgins, SC Benjamin, TM Stace, GJ Milburn, Brendon William Lovett, and EM Gauger, "Superabsorption of light via quantum engineering," Nature communications **5**, 4705 (2014).
[23] Giuseppe L Celardo, Fausto Borgonovi, Marco Merkli, Vladimir I Tsifrinovich, and Gennady P Berman, "Superradiance transition in photosynthetic light-harvesting complexes," The Journal of Physical Chemistry C **116**, 22105–22111 (2012).
[24] Heechul Park, Nimrod Heldman, Patrick Rebentrost, Luigi Abbondanza, Alessandro Iagatti, Andrea Alessi, Barbara Patrizi, Mario Salvalaggio, Laura Bussotti, Masoud Mohseni, *et al.*, "Enhanced energy transport in genetically engineered excitonic networks," Nature materials **15**, 211 (2016).
[25] Amir O Caldeira and Anthony J Leggett, "Path integral approach to quantum brownian motion," Physica A: Statistical mechanics and its Applications **121**, 587–616 (1983).
[26] Amir O Caldeira and Anthony J Leggett, "Quantum tunnelling in a dissipative system," Annals of Physics **149**, 374 – 456 (1983).
[27] Doron Cohen, "Unified model for the study of diffusion localization and dissipation," Physical Review E **55**, 1422 (1997).
[28] Massimiliano Esposito and Pierre Gaspard, "Emergence of diffusion in finite quantum systems," Journal of statistical physics **121**, 463–496 (2005).
[29] Freeman J. Dyson, "The dynamics of a disordered linear chain," Phys. Rev. **92**, 1331–1338 (1953).
[30] Ya. G. Sinai, "The limiting behavior of a one-dimensional random walk in a random medium," Theory of Probability & Its Applications **27**, 256–268 (1983).
[31] B Derrida and Ya Pomeau, "Classical diffusion on a random chain," Physical Review Letters **48**, 627 (1982).
[32] Bernard Derrida, "Velocity and diffusion constant of a periodic one-dimensional hopping model," Journal of Statistical Physics **31**, 433–450 (1983).
[33] Shlomo Havlin and Daniel Ben-Avraham, "Diffusion in disordered media," Advances in Physics **36**, 695–798 (1987).
[34] J-Ph Bouchaud, A Comtet, A Georges, and P Le Doussal, "Classical diffusion of a particle in a one-dimensional random force field," Annals of Physics **201**, 285–341 (1990).
[35] Jean-Philippe Bouchaud and Antoine Georges, "Anomalous diffusion in disordered media: Statistical mechanisms, models and physical applications," Physics Reports **195**, 127 – 293 (1990).
[36] Daniel Hurowitz and Doron Cohen, "Percolation, sliding, localization and relaxation in topologically closed circuits," Scientific reports **6** (2016).
[37] Daniel Hurowitz and Doron Cohen, "Relaxation rate of a stochastic spreading process in a closed ring," Phys. Rev. E **93**, 062143 (2016).
[38] Naomichi Hatano and David R. Nelson, "Localization transitions in non-hermitian quantum mechanics," Phys. Rev. Lett. **77**, 570–573 (1996).
[39] Naomichi Hatano and David R. Nelson, "Vortex pinning and non-hermitian quantum mechanics," Phys. Rev. B **56**, 8651–8673 (1997).
[40] Naomichi Hatano, "Localization in non-hermitian quantum mechanics and flux-line pinning in superconductors," Physica A: Statistical Mechanics and its Applications **254**, 317–331 (1998).
[41] David K. Lubensky and David R. Nelson, "Pulling pinned polymers and unzipping dna," Physical review letters **85**, 1572–5 (2000).
[42] David K. Lubensky and David R. Nelson, "Single molecule statistics and the polynucleotide unzipping transition," Phys. Rev. E **65**, 031917 (2002).
[43] Ariel Amir, Naomichi Hatano, and David R Nelson, "Localization in non-hermitian chains with excitatory/inhibitory connections," arXiv preprint arXiv:1512.05478 (2015).
[44] Ariel Amir, Naomichi Hatano, and David R Nelson, "Non-hermitian localization in biological networks," Physical Review E **93**, 042310 (2016).
[45] Karin A. Dahmen, David R. Nelson, and Nadav M. Shnerb, "Population dynamics and non-Hermitian localization," in *Statistical Mechanics of Biocomplexity*, edited by D. Reguera, J.M.G. Vilar, and J.M. Rubí (Springer Berlin Heidelberg, Berlin, Heidelberg, 1999) pp. 124–151.
[46] Ángel Rivas and Susana F Huelga, *Open Quantum Systems* (Springer, 2012).
[47] Dekel Shapira and Doron Cohen, In preparation.
[48] Vincent Hakim and Vinay Ambegaokar, "Quantum theory of a free particle interacting with a linearly dissipative environment," Phys. Rev. A **32**, 423–434 (1985).
[49] A. J. Leggett, S. Chakravarty, A. T. Dorsey, Matthew P. A. Fisher, Anupam Garg, and W. Zwerger, "Dynamics of the dissipative two-state system," Rev. Mod. Phys. **59**, 1–85 (1987).
[50] C Aslangul, N Pottier, and D Saint-James, "Quantum ohmic dissipation: cross-over between quantum tunnelling and thermally resisted motion in a biased tight-binding lattice," Journal de Physique **47**, 1671–1685 (1986).
[51] Aslangul, C., Pottier, N., and Saint-James, D., "Quantum brownian motion in a periodic potential: a pedestrian approach," J. Phys. France **48**, 1093–1110 (1987).
[52] Matthew P. A. Fisher and Wilhelm Zwerger, "Quantum brownian motion in a periodic potential," Phys. Rev. B **32**, 6190–6206 (1985).

[53] Julian Schwinger, "Brownian motion of a quantum oscillator," Journal of Mathematical Physics **2**, 407–432 (1961).
[54] Hermann Grabert, Peter Schramm, and Gert-Ludwig Ingold, "Quantum brownian motion: The functional integral approach," Physics Reports **168**, 115 – 207 (1988).
[55] Peter Hnggi and Gert-Ludwig Ingold, "Fundamental aspects of quantum brownian motion," Chaos: An Interdisciplinary Journal of Nonlinear Science **15**, 026105 (2005).
[56] Albert Schmid, "Diffusion and localization in a dissipative quantum system," Physical Review Letters **51**, 1506 (1983).
[57] Ulrich Weiss, Maura Sassetti, Thomas Negele, and Matthias Wollensak, "Dissipative quantum dynamics in a multiwell system," Zeitschrift für Physik B Condensed Matter **84**, 471–482 (1991).
[58] Marko Žnidarič, "Exact solution for a diffusive nonequilibrium steady state of an open quantum chain," Journal of Statistical Mechanics: Theory and Experiment **2010**, L05002 (2010).
[59] Viktor Eisler, "Crossover between ballistic and diffusive transport: the quantum exclusion process," Journal of Statistical Mechanics: Theory and Experiment **2011**, P06007 (2011).
[60] Marko Žnidarič and Martin Horvat, "Transport in a disordered tight-binding chain with dephasing," The European Physical Journal B **86**, 67 (2013).
[61] Xizhi Han and Sean A. Hartnoll, "Locality bound for dissipative quantum transport," Phys. Rev. Lett. **121**, 170601 (2018).
[62] Heinz-Peter Breuer and Francesco Petruccione, *The theory of open quantum systems* (Oxford University Press on Demand, 2002).
[63] Edward Brian Davies, *Quantum theory of open systems* (Academic Press, 1976)
